# Supplementary material for: ATG9A vesicles are a subtype of intracellular nanovesicle
Source: J Cell Sci. 2025 Apr 9;138(7):jcs263852. doi: 10.1242/jcs.263852 (PMC12045599; doi:10.1242/jcs.263852)
Supplement: Supplementary information [file joces-138-263852-s1.pdf]

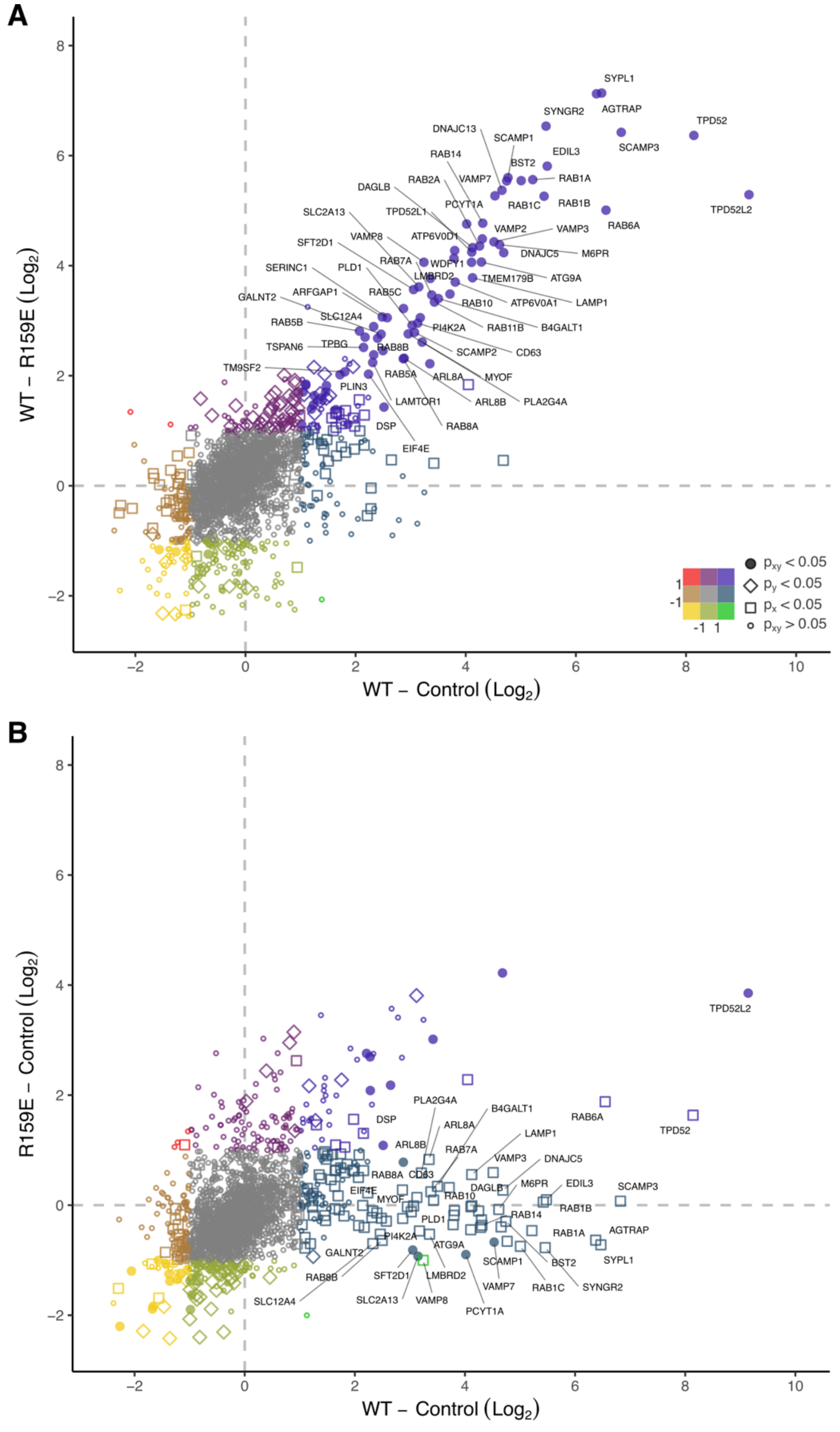

Fig. S1. Verifying the INV proteome. (A,B) INV protein enrichment data. Data from Fig. 1 replotted to compare enrichment in WT vs R159E against WT vs Control pulldown (A), or R159E vs Control against WT vs Control pulldown (B). Proteins significantly enriched in WT vs Control are labeled sparsely. Colors indicate the fold-enrichment (log2) while shapes indicate p-values of the comparison, as indicated.

Journal of Cell Science • Supplementary information

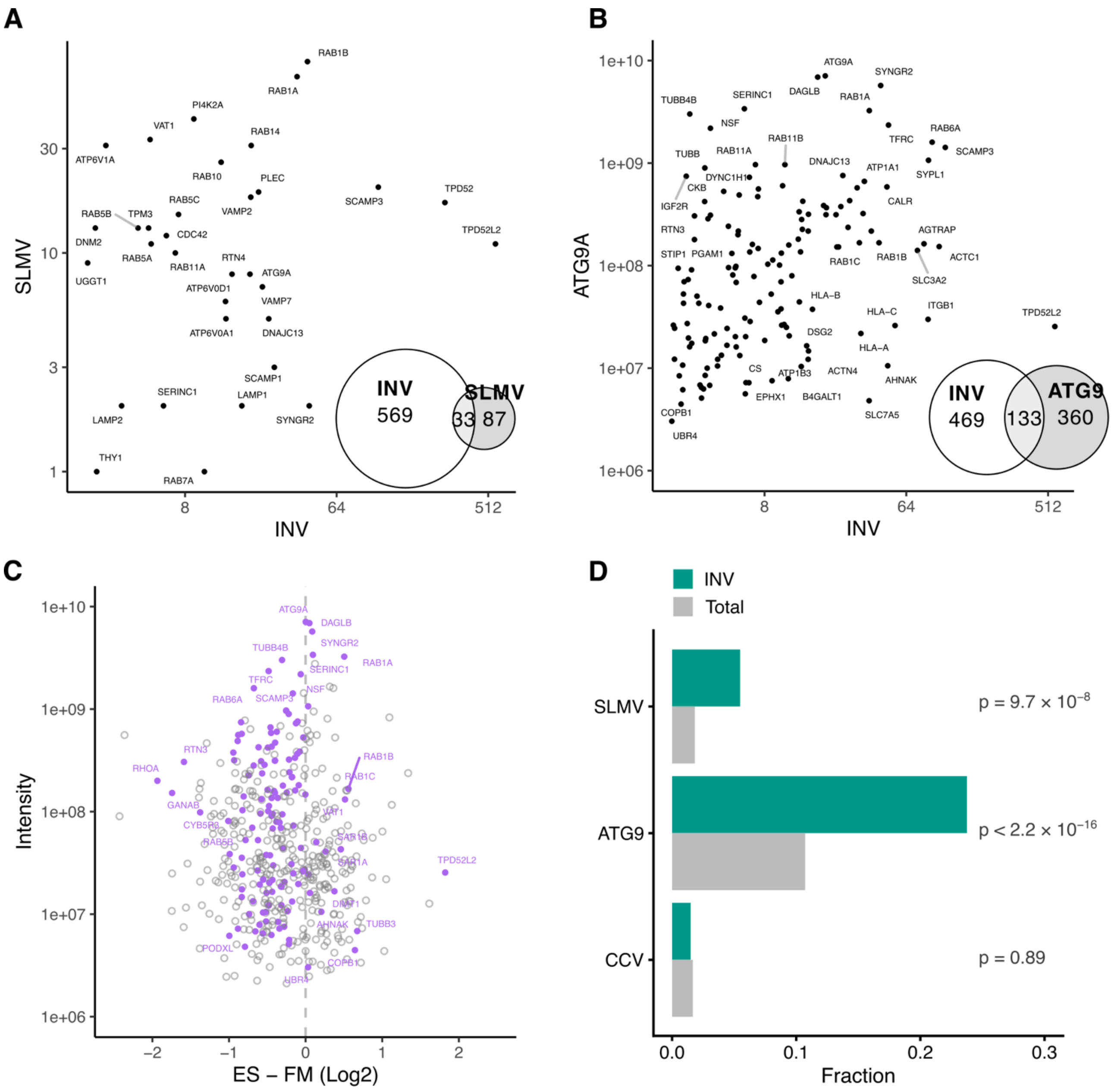

Fig. S2. Comparison of the INV proteome with published vesicle datasets. (A) Comparison of INV enrichment data with SLMV proteomic dataset (Salazar et al., 2005). Log-log plot of SMLV peptide values vs INV enrichment data. (B) Comparison of INV enrichment data with ATG9 proteomic dataset (Judith et al., 2019). Log-log plot of ATG9 intensity values *vs* INV enrichment data. Insets: Euler plot to show the the number of proteins in each dataset and their overlap. (C) Proteins associated with ATG9A-positive membranes from cells incubated in full media (FM) or EBSS for amino acid depletion (ES). Proteins that are enriched in the INV dataset are highlighted purple. (D) Fractions of INV proteins and of total proteins that were featured in the indicated dataset. Enrichment of dataset proteins in INVs versus total is indicated, p-values from Pearson's Chi-squared test.

Journal of Cell Science • Supplementary information

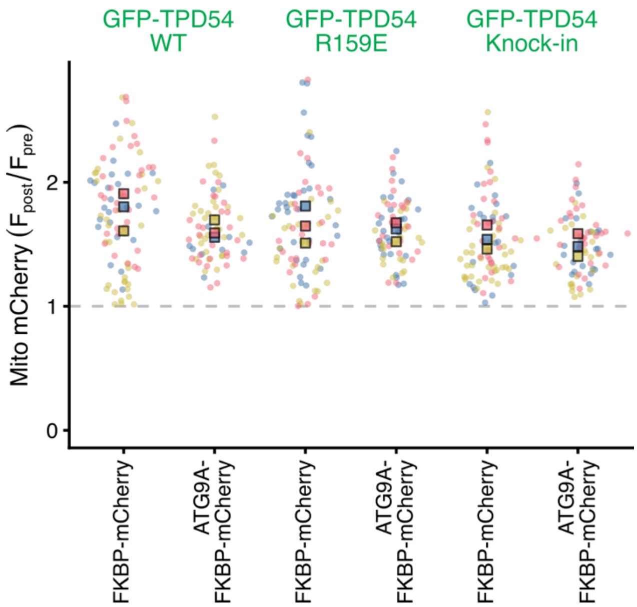

Fig. S3. Relocalization of FKBP-mCherry or ATG9A-FKBP-mCherry. Superplot to show the ratio of mitochondrial fluorescence of FKBP-mCherry or ATG9A-FKBP-mCherry post- vs pre-rapalog treatment. Spots indicate individual cell measurements (n = 21 - 69 per repeat), colors indicate independent experimental repeats (n = 3), squares show the mean value for each replicate. Co-relocation results for the green channel are shown in Fig. 4.

Journal of Cell Science • Supplementary information

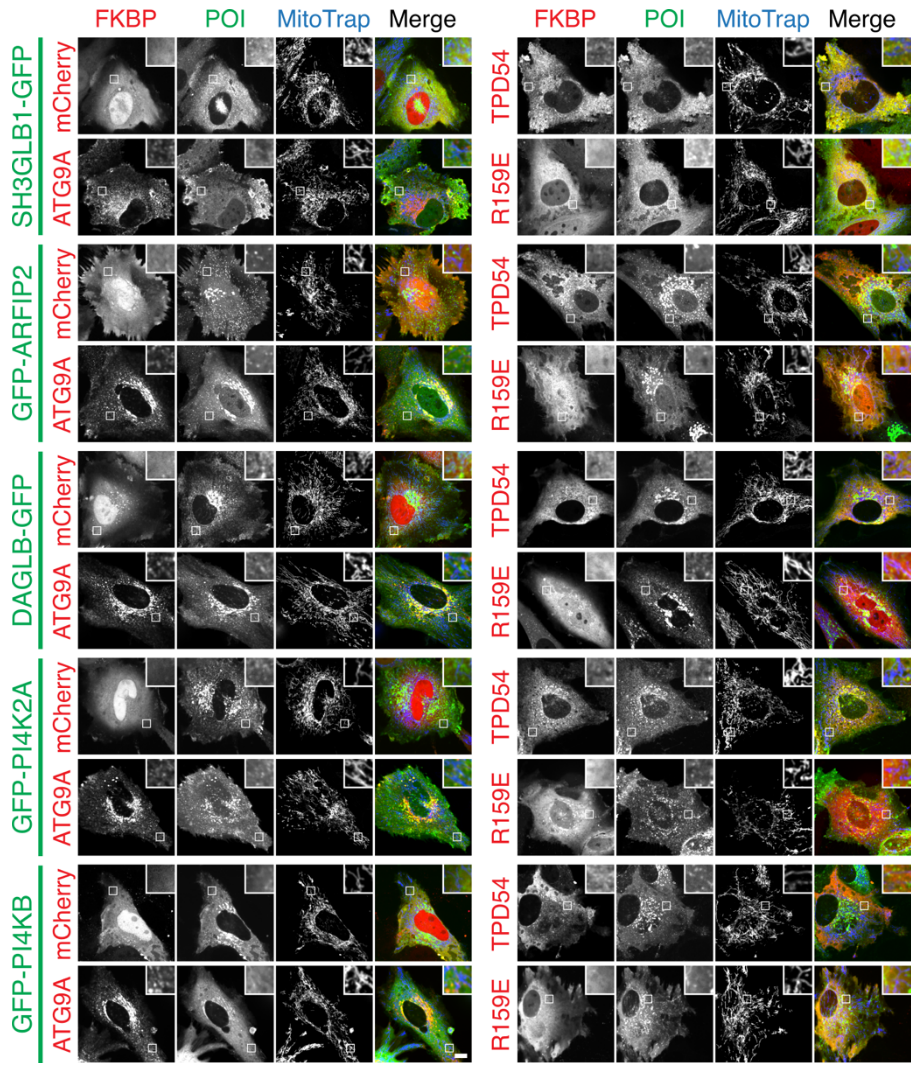

Fig. S4. ATG9A-flavor INVs have ATG9 vesicle cargos – pre-rapalog images. (A) Representative confocal images of HeLa cells expressing MitoTrap (pMito-EBFP2-FRB T2098L, blue) and either FKBP-mCherry, ATG9A-FKBP-mCherry, mCherry-FKBP-TPD54 WT, or mCherry-FKBP-TPD54 R159E (red). Cells are co-expressing GFP-tagged SH3GLB1, ARFIP2, DAGLB, PI4K2A, or PI4KB as indicated (green). For each condition, only the pre-treatment image is shown, for the post-rapalog images see Fig. 6. Scale bar, 10 μm; insets, 4 × zoom.

Journal of Cell Science • Supplementary information

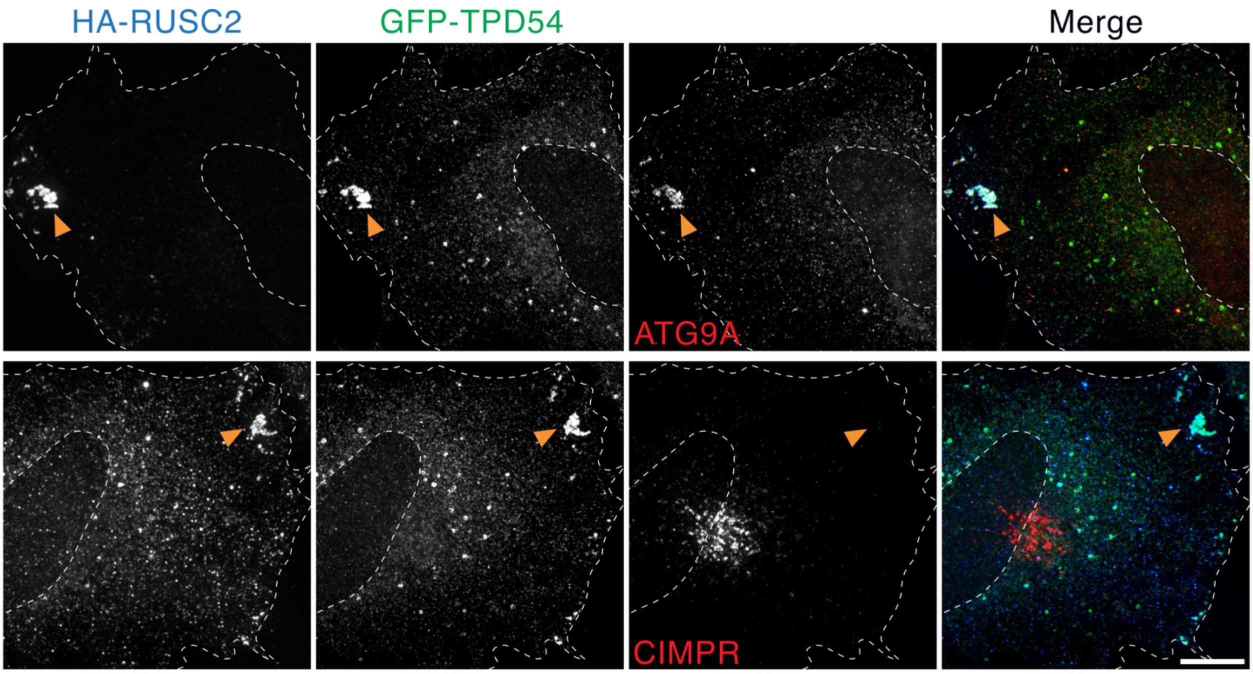

Fig. S5. Selective relocalization of ATG9A-flavor INVs using RUSC2. Representative confocal micrographs of GFP-TPD54 (green) knock-in HeLa cells overexpressing HA-RUSC2 (blue). Cells were fixed and stained for ATG9A or CIMPR (red, as indicated), anti-HA as well as GFP boost. Orange arrowheads indicate accumulation of vesicular material at the cell periphery. Scale bar, 10 μm.

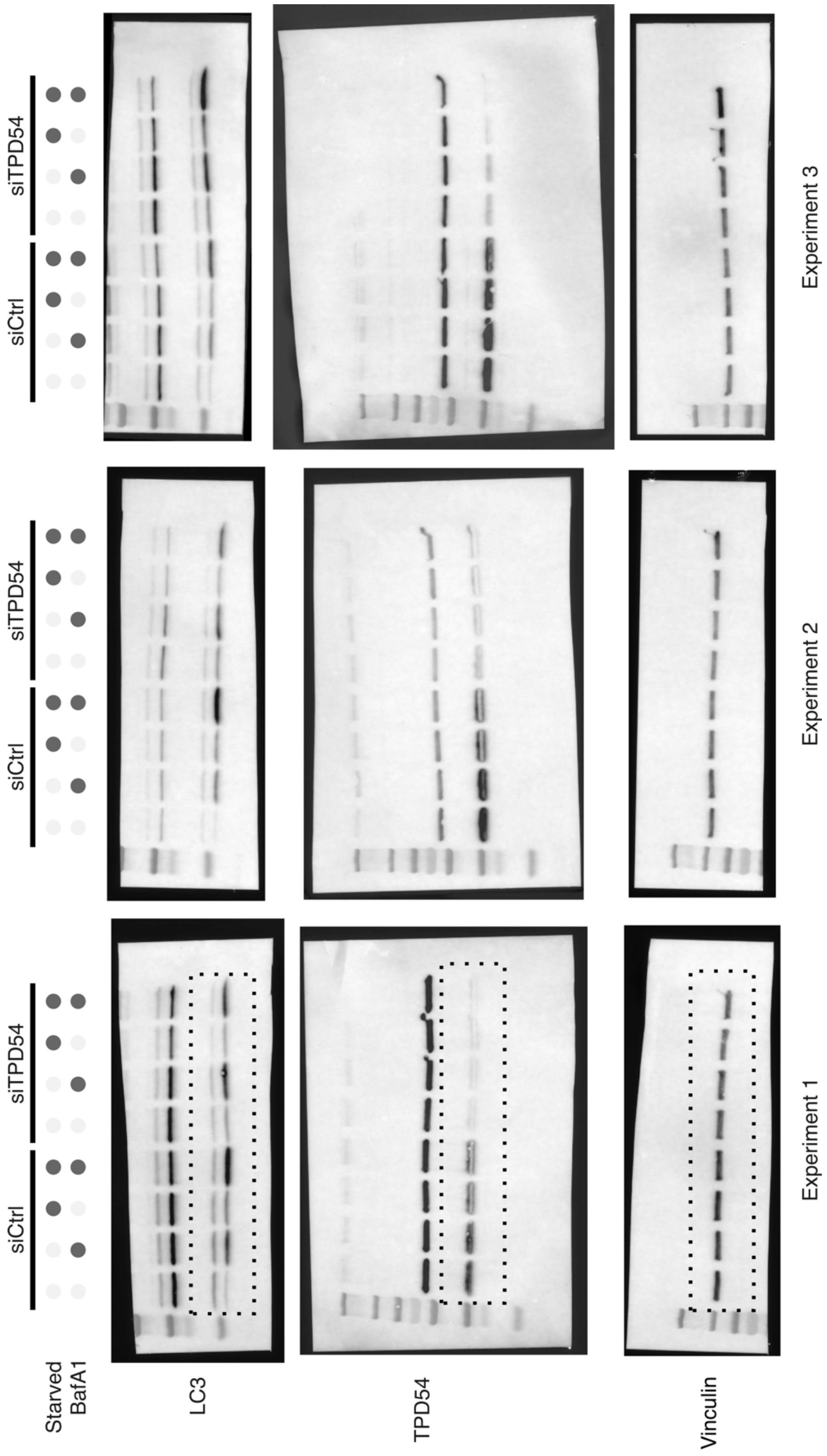

Fig. S6. Effect of TPD54 depletion on LC3 lipidation under starvation. Three experiments where cells were transfected with siCtrl or siTPD54, starved (3 h) or not (fed) and either treated with BafA1 (100 μM) or not (DMSO), as indicated. LC3 was detected with anti-LC3B antibody, endogenous TPD54 was detected using an anti-TPD54 antibody; detection of Vinculin was used as a loading control. Dotted lines indicate the crops shown in Fig. 7.

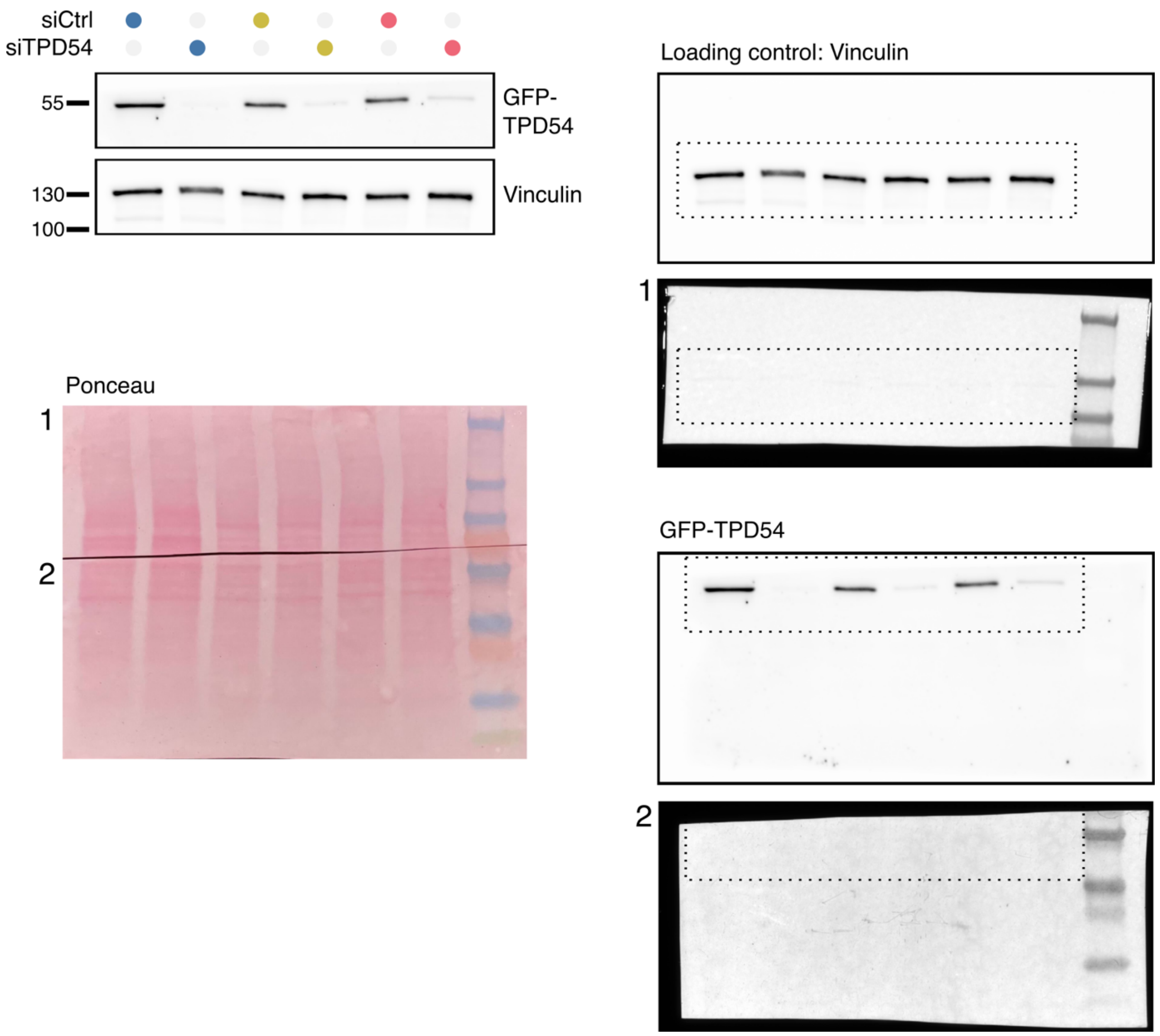

Fig. S7. Depletion of TPD54 using RNAi. Western blot to show knock-down of TPD54 in the experiments shown in Figs 7 and 8). Endogenous GFP-TPD54 was detected using an anti-GFP antibody and vinculin was used as a loading control. Markers in kDa, colors indicate experimental repeats in Figs 7 and 8) and siRNA treatment. Other panels show the full Ponceau stained membrane and the full imaged area.

Journal of Cell Science • Supplementary information

Table S1. The INV proteome. A consolidated list of INV proteins determined by proteomics ranked by their fold enrichment over control. 602 proteins that had a fold change of > 2 and p < 0.05 are included. The enrichment in WT, R159E and/or knock-in over their respective controls is indicated as WT, R159E, and/or INV, respectively.

Available for download at
https://journals.biologists.com/jcs/article-lookup/doi/10.1242/jcs.263852#supplementary-data

Table S2. PANTHER protein classification of INV proteins. The consolidated INV proteome with assigned class and subclass from PANTHER protein classification are shown ordered by class-subclass.

Available for download at
https://journals.biologists.com/jcs/article-lookup/doi/10.1242/jcs.263852#supplementary-data

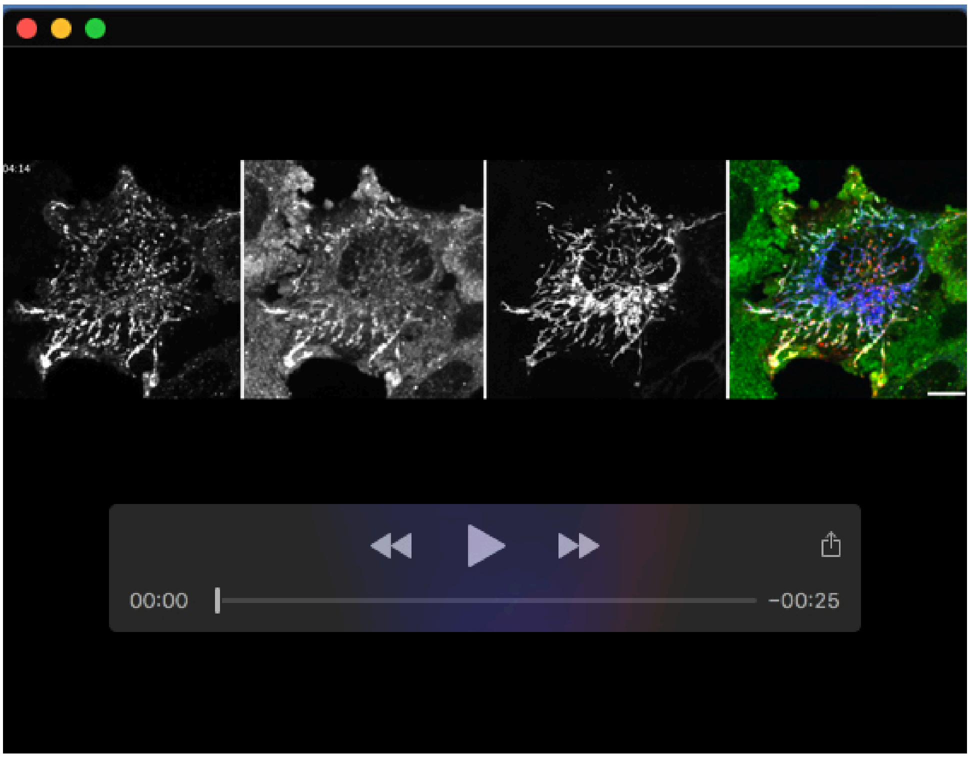

Movie 1. Relocalization of ATG9A-FKBP-mCherry causes co-relocation of endogenous GFP-TPD54. Movie of GFP-TPD54 (panel 2, green) knock-in cells expressing ATG9A-FKBP-mCherry (panel 1, red) and MitoTrap (panel 3, blue); capture of ATG9A-positive vesicles at the mitochondria is induced by rapalog (5 μM at 40 s). Time, mm:ss. Playback, 10 fps. Scale bar, 10 μm.
