# Supplementary material for: ATG9A vesicles are a subtype of intracellular nanovesicle
Source: J Cell Sci. 2025 Apr 9;138(7):jcs263852. doi: 10.1242/jcs.263852 (PMC12045599; doi:10.1242/jcs.263852)
Supplement: Supplementary file 1 [file joces-138-263852_review_history.pdf]

Journal of Cell Science

ATG9A vesicles are a subtype of intracellular nanovesicle

Mary Fesenko, Daniel J Moore, Peyton Ewbank, Stephen J Royle, Elizabeth Courthold
DOI: 10.1242/jcs.263852

Editor: Robert Parton

Review timeline

Original submission:	15 January 2025
Editorial decision:	13 February 2025
First revision received:	25 February 2025
Accepted:	27 February 2025

Original submission

First decision letter

MS ID#: jcs.263852

MS TITLE: ATG9A vesicles are a subtype of intracellular nanovesicle

AUTHORS: Mary Fesenko; Daniel J Moore; Peyton Ewbank; Stephen J Royle; Elizabeth Courthold
ARTICLE TYPE: Research Article

We have now reached a decision on the above manuscript.

To see the reviewers' reports and a copy of this decision letter, please go to:

As you will see, the reviewers raise a number of concerns that prevent me from accepting the paper at this stage. I hope that you can address these issues in full and I look forward to receiving a revised manuscript in due course.

Reviewer 1

Advance summary and potential significance to field

Using advanced technologies, the authors of this study provides a better compositional and structural characterization of the ATG9 vesicles, which are key for autophagosome formation during autophagy. They also identify TDP54 as a component of these vesicles and show that it plays a role in ATG9A sorting from the TGN/Golgi and thus is also important for normal progression of autophagy.

Comments for the author

MAJOR CRITICISMS

The term ATG9 vesicles is confusing. Although it has been used in rare cases, these vesicles are typically referred to as ATG9A vesicles (or ATG9A-positive vesicles, ATG9A-containing vesicles, also with the suffix Atg9/ATG9A). As the proteomics analysis by the authors shows, the isolated vesicles do not contain ATG9B; therefore, defining them as ATG9 alone is incorrect and could lead to confusion. Furthermore, the authors provide a better morphological and compositional characterization of the Atg9/ATG9A-positive vesicles and have chosen to rename them ATG9A-

© 2025. Published by The Company of Biologists under the terms of the Creative Commons Attribution License (https://creativecommons.org/licenses/by/4.0/).

flavored INVs, suggesting that they represent a novel or distinct structure compared to the Atg9/ATG9A-positive vesicles (although they conclude themselves that they are the same structure). This is both confusing and incorrect, considering the body of work by other researchers in the field who have referred to these carriers as Atg9/ATG9A-positive vesicles for the past two decades. It would be more accurate to state that the Atg9/ATG9A-positive vesicles are a subtype or subset of INVs, without renaming them (throughout the manuscript). Additionally, substantial work has been done on Atg9/ATG9A-positive vesicles in yeast, which the authors have completely disregarded. This includes solid evidence from the Ohsumi lab showing that these carriers are nanovesicles and from several laboratories that they serve as one of the seed/donor membranes for the phagophore formation (and perturbing their traffick impair autophagy). This literature must be discussed and cited.

Overall, the first part of the manuscript provides a better characterization of Atg9/ATG9A-positive vesicles, but it does not introduce new information about their morphology or function. It also confirms the presence of other proteins previously identified by others. The completely novel aspect of this manuscript is the identification of TPD54 as a new autophagy player, with a specific role in the trafficking of Atg9/ATG9A-positive vesicles.

Fig. 8: AP-4 depletion must be used as a control

The authors must examine by immunoprecipitation the interaction between ATG9A, AP-4 and TDP54, in the presence or the absence of AP-4 or TDP54. This will provide some mechanistic advances to the study.

MINOR POINTS

There have been a few studies that examined Atg9/ATG9A-positive vesicles at the ultrastructural level using electron microscopy. How do the authors' findings, which suggest that these carriers are INVs, relate to these studies? Do they lead to the same conclusions? If not, please explain any differences.

The author may consider to discuss yeast Atg23 as a counterpart of TDP54, because involved in Atg9 sorting from the Golgi and also a small peripheral membrane associated protein.

<u>Reviewer 2</u>

Advance summary and potential significance to field

In this manuscript Fesenko and colleagues further characterize a class of very intriguing small non-coated vesicles called intracellular nanovesicles INVs. In this manuscript the authors define the proteome of INV via immuno-purification of vesicles from living cells. They show that INVs have a very heterogeneous composition with many cargoes and proteins involved in many different cellular processes. ATG9, a protein known to localise to Golgi-derived vesicles responsible for autophagosome formation, is highly enriched in the INVs proteome. The authors validate their finding by showing that ATG9 also re-localises to mitochondria when INVs are brought to mitochondria with chemical dimerizer using a re-localization assays well established in the Royle lab. Quantification of the re-localization experiments suggest that all ATG9 vesicles are a sub-type of INVs. This is further supported by the fact that proteins which have been identified as ATG9 vesicles residents also redistribute to mitochondria when the re-localization assay is performed. In a last set of experiments Fesenko and colleagues can show that inhibition of INVs via siRNA of TDP54 inhibits autophagic flux as shown by impaired LC3 lipidation, reduced LC3-positive punctae and inhibition of starvation-induced loss of ATG9-positive at the Golgi.

Overall, the paper is very well written, the data very well presented and analysed, and I only have a few minor comments can be addressed with minor text changes. I find the characterization of a type of INVs exciting and of interest of the broad membrane trafficking field.

Line 6 p2 "We combined the data from both approaches to obtain a list of 602 proteins that we consider a first INV proteome" I am curious to know why the authors combine the two proteomes

© 2025. Published by The Company of Biologists under the terms of the Creative Commons Attribution License (https://creativecommons.org/licenses/by/4.0/).

rather than further refining and looking at the hits highlighted in both datasets? I wonder how the proteome from overexpression compares to the proteome from endogenously tagged TPD54?

Line 54 p5 CCV is not defined

Fig 5E. I understand the train of thoughts of the authors here. But as data comes from fixed cell imaging with antibodies if I understand correctly have the authors considered that some of the cytoplasmic staining may be background? Why is the trapping of INV vesicles via TDP54 more efficient than the trapping via ATG9?

The authors maybe could add a sentence explaining how TDP54 depletion affects INVs/their function before they start explaining the effect of TDP54 on autophagic flux. From what I read in the discussion the function of TDP54 is unclear. But what does siRNA of TDP54 do to the whole pool of INVs?

First revision

Author response to reviewers' comments

Fesenko *et al*. Response to Reviewers

We thank the Reviewers for their valuable input on our work. Below is a point-by-point response to their comments.

Comments from the Reviewers:

Reviewer 1: SUMMARY OF THE ADVANCE MADE IN THIS PAPER AND ITS POTENTIAL SIGNIFICANCE TO THE FIELD

Using advanced technologies, the authors of this study provides a better compositional and structural characterization of the ATG9 vesicles, which are key for autophagosome formation during autophagy. They also identify TDP54 as a component of these vesicles and show that it plays a role in ATG9A sorting from the TGN/Golgi and thus is also important for normal progression of autophagy.

Reply: We thank the Reviewer for their comments on our paper and for taking the time to critique our work.

SUGGESTIONS TO AUTHORS

MAJOR CRITICISMS

The term ATG9 vesicles is confusing. Although it has been used in rare cases, these vesicles are typically referred to as ATG9A vesicles (or ATG9A-positive vesicles, ATG9A-containing vesicles, also with the suffix Atg9/ATG9A). As the proteomics analysis by the authors shows, the isolated vesicles do not contain ATG9B; therefore, defining them as ATG9 alone is incorrect and could lead to confusion.

Reply: We thank the Reviewer for this perspective. The term "ATG9 vesicles" is used in the title of two papers in our reference list, so it's not unconventional. It's a nice term because it reflects the heritage of these vesicles being discovered in yeast (Atg9 vesicles) as well as signifying mammalian work (ATG9A/ATG9B). The Reviewer is quite correct that ATG9B is absent from our work (its expression is tissue specific) so it would be more accurate to refer to them as ATG9A vesicles. **We have changed all instances in the paper.**

© 2025. Published by The Company of Biologists under the terms of the Creative Commons Attribution License (https://creativecommons.org/licenses/by/4.0/).

Furthermore, the authors provide a better morphological and compositional characterization of the Atg9/ATG9A-positive vesicles and have chosen to rename them ATG9A-flavored INVs, suggesting that they represent a novel or distinct structure compared to the Atg9/ATG9A-positive vesicles (although they conclude themselves that they are the same structure). This is both confusing and incorrect, considering the body of work by other researchers in the field who have referred to these carriers as Atg9/ATG9A-positive vesicles for the past two decades. It would be more accurate to state that the Atg9/ATG9A-positive vesicles are a subtype or subset of INVs, without renaming them (throughout the manuscript).

Reply: we disagree with this point. We do *not* suggest that ATG9A-flavored INVs represent a novel or distinct structure to ATG9A vesicles; that would indeed be confusing and incorrect. Our findings suggest that ATG9A vesicles are a subset of a much larger family of vesicles – Intracellular Nanovesicles. **We have retained the name ATG9A-flavor INVs in the paper** for two reasons. First, it's clear now from multiple studies that, in mammalian cells, 'small vesicles containing ATG9A' have a diverse range of functions. Their involvement in autophagy has been the most extensively characterized, but they were also shown to mediate plasma membrane repair, integrin recycling, lipid mobilization from lipid droplets among other cellular processes. Our work shows that these 'small vesicles containing ATG9A' are INVs. Therefore, they are better thought of as INVs with ATG9A-mediated lipid scramblase activity, hence we decided to refer to them as 'ATG9A-flavour INVs'. Second, a major goal of our lab is to define the functions of INVs, which are a large transport vesicle family that we discovered. We have found that 20% of them are ATG9A flavor and we are in the process of characterizing the other 80%. Our chosen terminology is useful to distinguish them from other flavors while we undertake this endeavor.
To be clear, while we feel that this name is a more accurate reflection of the functions of these vesicles in cells, and will be more helpful in the future, *we don't wish to impose a rebrand on anyone*. Scientists working on these vesicles will refer to them how they please.

Additionally, substantial work has been done on Atg9/ATG9A-positive vesicles in yeast, which the authors have completely disregarded. This includes solid evidence from the Ohsumi lab showing that these carriers are nanovesicles and from several laboratories that they serve as one of the seed/donor membranes for the phagophore formation (and perturbing their traffick impair autophagy). This literature must be discussed and cited.

Reply: We felt it was better to focus our discussion on the role of ATG9A vesicles in mammalian cells, as this is the system we work on. Moreover, TPD52-like proteins are specific to metazoans so it was hard for us to directly link our findings with those about Atg9 vesicles in yeast. Our manuscript already included citations of yeast work, however, we have taken the Reviewer's feedback on board and **have revised the paper** as follows.

In the Introduction, we added more references to yeast work and clear statement of the seminal work on Atg9 vesicles in yeast:

> Of the core set of Atg proteins that are essential for autophagy, Atg9/ATG9A is the only transmembrane protein (Noda et al., 2000; Nishimura and Tooze, 2020) and functions as a lipid scramblase (Maeda et al., 2020; Guardia et al., 2020; Matoba et al., 2020). Pioneering work in *S. cerevisiae* showed that Atg9 resides on 30-60 nm diameter vesicles derived from the Golgi which diffuse freely in the cytoplasm (Ohashi and Munro, 2010; Yamamoto et al., 2012; Mari et al., 2010). In mammalian cells, ATG9A is also found on small uncoated vesicles, termed 'ATG9A vesicles', that cycle between the *trans*-Golgi network, the plasma membrane and the endosomal system (Orsi et al., 2012; Popovic and Dikic, 2014; Young et al., 2006). During autophagy, ATG9A vesicles move to the phagophore assembly site, where they contribute to phagophore formation through mechanisms that are still being determined (Choi et al., 2024; Holzer et al., 2024). However, there is a growing consensus that both in yeast and mammalian systems Atg9/ATG9A vesicles function as the seed for the autophagosomal membrane and may also deliver additional lipids or protein cargoes to it through transient interactions at later stages during autophagosome biogenesis (Orsi et al., 2012; Karanasios et al., 2016; Olivas et al., 2023; Broadbent et al., 2023; Sawa-Makarska et al., 2020).

In the Discussion, a new paragraph detailing how our findings may translate to yeast:

> Atg9 vesicles were first described in *S. cerevisiae* as 30-40 nm diameter vesicles that diffuse freely in the cytoplasm (Mari et al., 2010; Yamamoto et al., 2012). INVs in mammalian cells are

© 2025. Published by The Company of Biologists under the terms of the Creative Commons Attribution License (https://creativecommons.org/licenses/by/4.0/).

similar in size and behavior (Larocque et al., 2020; Sittewelle and Royle, 2024). It is therefore tempting to extrapolate our findings to yeast and ask if they also have INVs. TPD52-like proteins are specific to metazoans and, since they are currently the only marker for INVs, yeast do not have these vesicles using this definition. On the other hand, yeast do not have an AP-4 complex either, yet AP-4 is crucial for ATG9A vesicle formation in mammals, so molecular differences between the two systems need not preclude functional homology between Atg9 vesicles and INVs. While yeast do not have TPD52-like proteins, Atg23, Atg11 and Atg17 are dimeric membrane tethers that are important for Atg9 vesicle function in autophagy (Hawkins et al., 2022; He et al., 2006; Sekito et al., 2009). These proteins do not have human homologs yet there is superficial structural similarity to TPD52-like proteins, and they could be investigated as distant functional homologs. Further work is needed to determine if Atg9 vesicles in yeast are also a subset of a larger vesicle family or if they are a discrete vesicle class specialized for autophagy.

Overall, the first part of the manuscript provides a better characterization of Atg9/ATG9A-positive vesicles, but it does not introduce new information about their morphology or function. It also confirms the presence of other proteins previously identified by others. The completely novel aspect of this manuscript is the identification of TPD54 as a new autophagy player, with a specific role in the trafficking of Atg9/ATG9A-positive vesicles.

Reply: this comment was disheartening since the Reviewer had not appreciated the broader membrane trafficking context of our work. However, it was useful in understanding how our work might be viewed by those looking solely through the lens of autophagy.

Fig. 8: AP-4 depletion must be used as a control

Reply: the two variables in this experiment – TPD54 expression and starvation – are controlled for by the GL2 siRNA and the fed condition, respectively. So there isn't a missing control in the experiment. We do not think that this experiment would be strengthened by the addition of this extra condition. First, the localization of ATG9A at the TGN following depletion of AP-4 has been investigated by Davies/Borner/Robinson and Bonifacino labs (Mattera et al., 2017; Davies et al., 2018). Second, the experiment was to test if INVs were involved in the phenomenon of starvation-induced ATG9A redistribution. Third, we didn't observe AP-4 subunits in our proteome, so it wouldn't strengthen the core message of our paper, which is to show that ATG9A vesicles are a subset of INVs that we observed proteomically.

The authors must examine by immunoprecipitation the interaction between ATG9A, AP-4 and TDP54, in the presence or the absence of AP-4 or TDP54. This will provide some mechanistic advances to the study.

Reply: this suggestion is beyond the scope of the current work. The scope, as defined by the title, is supported by the work currently in the paper. The question of whether AP-4 is involved in the formation of ATG9A vesicles has already been addressed by Davies/Borner/Robinson and Bonifacino labs and our RUSC2 experiment (Supplementary Fig S5) is entirely in keeping with that work. Whether or not AP-4 is involved in the formation of ATG9A-flavor INVs, is not central to our characterization of these vesicles. The suggested experiments would require three different approaches to purification while we currently only have one working and to isolate three different classes of vesicles and test for other components following depletion, would be a substantial undertaking. At best, it would confirm what is already reported.

MINOR POINTS

There have been a few studies that examined Atg9/ATG9A-positive vesicles at the ultrastructural level using electron microscopy. How do the authors' findings, which suggest that these carriers are INVs, relate to these studies? Do they lead to the same conclusions? If not, please explain any differences.

© 2025. Published by The Company of Biologists under the terms of the Creative Commons Attribution License (https://creativecommons.org/licenses/by/4.0/).

Reply: we thank the Reviewer for this great point. They are very similar. In adding more text about yeast Atg9 vesicles (see point above) we are now able to make this point more clearly.

The author may consider to discuss yeast Atg23 as a counterpart of TDP54, because involved in Atg9 sorting from the Golgi and also a small peripheral membrane associated protein.

Reply: again, this was a great suggestion. We had not seen the work on Atg23 nor that on Atg11 and Atg17, which perform similar functions. As described above, we now have a paragraph in the Discussion to talk about how our findings can be extrapolated to yeast and we now specifically mention these proteins.
Briefly, like TPD54, they dimerize, have coiled-coils and/or amphipathic helices, and are membrane-associated. Their predicted structures do not strongly suggest structural homology. Nonetheless, we now extend our Discussion to consider our work in the context of yeast Atg9 vesicles and we take on board this great suggestion as a direction for future work.

Reviewer 2: In this manuscript Fesenko and colleagues further characterize a class of very intriguing small non-coated vesicles called intracellular nanovesicles INVs. In this manuscript the authors define the proteome of INV via immuno-purification of vesicles from living cells. They show that INVs have a very heterogeneous composition with many cargoes and proteins involved in many different cellular processes. ATG9, a protein known to localise to Golgi-derived vesicles responsible for autophagosome formation, is highly enriched in the INVs proteome. The authors validate their finding by showing that ATG9 also re-localises to mitochondria when INVs are brought to mitochondria with chemical dimerizer using a re-localization assays well established in the Royle lab. Quantification of the re-localization experiments suggest that all ATG9 vesicles are a sub-type of INVs. This is further supported by the fact that proteins which have been identified as ATG9 vesicles residents also redistribute to mitochondria when the re-localization assay is performed. In a last set of experiments Fesenko and colleagues can show that inhibition of INVs via siRNA of TDP54 inhibits autophagic flux as shown by impaired LC3 lipidation, reduced LC3-positive punctae and inhibition of starvation-induced loss of ATG9-positive at the Golgi.

Overall, the paper is very well written, the data very well presented and analysed, and I only have a few minor comments can be addressed with minor text changes. I find the characterization of a type of INVs exciting and of interest of the broad membrane trafficking field.

Reply: We thank the Reviewer for their time evaluating our manuscript and we very much appreciate their positive comments on our work.

Line 6 p2 "We combined the data from both approaches to obtain a list of 602 proteins that we consider a first INV proteome" I am curious to know why the authors combine the two proteomes rather than further refining and looking at the hits highlighted in both datasets? I wonder how the proteome from overexpression compares to the proteome from endogenously tagged TPD54?

Reply: the two approaches produced datasets that differed. Broadly, the first proteome (with overexpression) had fewer hits with very high confidence, whereas the second (at endogenous level) was the converse. Our initial goal was, as the reviewer suggests, to home in on the core INV components common to both. However, we realised through analysing the different repeats, that the proteome was not a fixed list, but a changing cast of cargos being trafficked at the time of the experiment. We wanted to reflect the heterogeneity seen in this class of vesicle and so the approach of combining both datasets seemed the best way to collate a useful resource for people in the field to use in the future. In the supplementary information, we have annotated whether each INV protein was in both datasets or just one dataset, for anyone interested in this question.

Line 54 p5 CCV is not defined

Reply: **Definition added**.

© 2025. Published by The Company of Biologists under the terms of the Creative Commons Attribution License (https://creativecommons.org/licenses/by/4.0/).

Fig 5E. I understand the train of thoughts of the authors here. But as data comes from fixed cell imaging with antibodies if I understand correctly have the authors considered that some of the cytoplasmic staining may be background? Why is the trapping of INV vesicles via TDP54 more efficient than the trapping via ATG9?

Reply: The detection of ATG9A following relocalization of TPD54 is done with direct immunofluorescence, i.e. the ATG9A antibody has a fluorophore directly conjugated to it. This reduces the amount of background in the experiment considerably (see Fig 3B for example). Moreover, because we see similar loss following relocalization of tagged ATG9A where there is no cytoplasmic staining, we think that the contribution of a background signal must be minimal. We believe that the signal that cannot be relocalized is mainly TGN-resident ATG9A, which is not excluded from the region in which we measure the cytoplasmic signal. And that this difference explains the difference in efficiency of capture.

We appreciate the comment that the Reviewer understood the train of thought here. This is a key point in the paper and it is quite difficult to explain, so we're glad that we succeeded in conveying it.

The authors maybe could add a sentence explaining how TDP54 depletion affects INVs/their function before they start explaining the effect of TDP54 on autophagic flux. From what I read in the discussion the function of TDP54 is unclear. But what does siRNA of TDP54 do to the whole pool of INVs?

Reply: It is hard for us to be definitive, because we are still investigating the function of TPD54 and the effect of its loss on the whole pool of INVs. In response to this comment, **we have changed this part** to:

> ATG9A has been shown to be redistributed from the TGN upon starvation (Orsi et al., 2012). We therefore tested whether INVs were involved in this phenomenon by examining the effect of TPD54 depletion on starvation-induced ATG9A redistribution. Depletion of TPD54 has previously been shown to inhibit INV-mediated traffic on anterograde and recycling pathways (Larocque et al., 2020). Using immunofluorescence...

Second decision letter

MS ID#: jcs.263852R1

MS TITLE: ATG9A vesicles are a subtype of intracellular nanovesicle

AUTHORS: Mary Fesenko; Daniel J Moore; Peyton Ewbank; Stephen J Royle; Elizabeth Courthold
Article Type: Research Article

I am happy to tell you that your manuscript has been accepted for publication in Journal of Cell Science, pending standard publication integrity checks.

© 2025. Published by The Company of Biologists under the terms of the Creative Commons Attribution License (https://creativecommons.org/licenses/by/4.0/).
